# Supplementary material for: Factors influencing the acceptability of alcohol drinking for a patient with colorectal cancer
Source: PLoS One. 2023 Dec 28;18(12):e0296409. doi: 10.1371/journal.pone.0296409 (PMC10754451; doi:10.1371/journal.pone.0296409)
Supplement: Attachment — Submitted filename: Response to Reviewers.docx [file pone.0296409.s001.docx]

Response to Reviewers

We thank very much the Academic Editor and the Reviewers for their relevant and helpful comments, which we believe have improved our manuscript’s quality. Please find below a document that responds to each point raised by the Academic Editor and Reviewers.

Reviewer 1

1. When presenting the rationale of the study the authors describe possible consequences of stigma on persons living with colorectal cancer. However, as a reader, I would also like to know the beliefs and thoughts underlying stigma attitudes towards those patients and how is this stigma expressed.

*Response: We thank Reviewer 1 for this relevant comment that helped us improving the manuscript. We thus have added three sentences and references on beliefs and thoughts that underlie stigmatizing attitudes towards patients with colorectal cancer (lines 74 to 79).*

2. The way the authors presented the theoretical framework is misleading: Functional Theory of Cognition is not a “method enabling to identify variables involved in one’s judgment”, but rather a theory of how individuals process information to form judgments.

Reference : Anderson NH. A functional theory of cognition. Psychology Press; 2014.

*Response: We thank Reviewer 1 for this clarification on the Functional Theory of Cognition. This part was modified accordingly (lines 106 to 108).*

3. Page 4 Line 100-102 : “The scenarios were based on four "within subject factors" supposed to have an impact on the perception of a person who drinks alcoholic beverages and who is diagnosed with colorectal cancer”.

Vignette-based methods require that factor selection for the scenarios should be based on evidence, not supposition. No previous research suggested that factors selected by the authors--“Drinking habits”, “Drinking behavior”, “Type of cancer diagnosis and prognosis” and level of “Physical activity” -- influence attitudes towards cancer patients with alcohol consumption habits.

*Response: We are grateful to Reviewer 1 for this valuable comment. In this manuscript’s new version, we have developed the rationale regarding the choice of factors for the construction of the scenarios (lines 119 to 122 and 126 to 135).*

4. The authors should provide information regarding recruitment of the participants: Where was the sample of health professionals recruited? Where was the sample of lay people recruited? Where were the participants approached? What was the recruitment procedure for each sample?

*Response: We have added information regarding the recruitment of participants, in the Participants section (lines 146 to 149).*

5. The research material is unclear. For instance, the understanding of the expression “drinks of alcohol beverages” in the vignette is confusing. Assessment of alcohol consumption cannot be based on the number of drinks solely. One important factor is the alcohol level in the beverage. 3 glasses of beer do not equal 3 glasses of whisky when assessing alcohol consumption.

*Response: We agree with this comment, even though it would have been difficult to take into account the type of alcoholic beverage, the volume taken and the percentage of alcohol in the drink. We now mention this aspect as a limitation (lines 376 to 378).*

6. The authors developed the rationale of their study and the discussion of their findings around stigma. However, their research did not investigate the issue of stigma. Their research question has focused on acceptability. Stigma and acceptability are two very different concepts.

*Response : We are grateful to Reviewer 1 for this comment. Indeed, stigma and acceptability are different concepts. To clarify this, we have further developed the definition of stigma in the Introduction section and clarified the relationship between stigma and acceptability (line 68 to 72). In addition, for improved accuracy of the text, the term “stigmatization” was changed to “acceptability judgments” in the results section.*

Reviewer 2

Please find the comments below:

1. I would highly recommend the authors to include 'alcohol drinking' instead of 'drinking' in the title to increase the visibility.

*Response: We thank Reviewer 2 for this valuable suggestion. The title and short title were modified accordingly.*

2. The authors have taken two type population - community persons and health professionals. I am very interested to know if there is any difference in the acceptability judgment between highly educated and low/no educated person among these population. (Editor: please clarify or add to the present paper)

*Response: We thank Reviewer 2 for this suggestion. We have now included the results regarding the influence of education level on acceptability judgments in each sample. These results are presented within the last paragraph of the sub-section entitled "Participants’ characteristics and descriptive statistics" in the Results section (lines 232 to 241).*

3. Did the authors find any difference in the acceptability judgment between male and female study participants? (Editor: please clarify or add to the present paper)

*Response: We apologize for this oversight. We now include t-test results regarding the lack of statistical significance of differences of acceptability judgements between male and female (lines 228 and 231).*
